# The triglyceride-glucose index is associated with coronary plaque features and clinical outcomes in patients with ST-segment elevation myocardial infarction

**DOI:** 10.3389/fendo.2025.1665292

**Published:** 2025-09-17

**Authors:** Yibo Guo, Lina Cui, Jiaqing Guo, Chengmei Jin, Lili Xiu, Yubo Gao, Chen Zhao, Xueming Xu, Jinfeng Tan, Jincheng Han, Lulu Li, Tao Chen, Jiannan Dai, Bo Yu, Chao Fang

**Affiliations:** ^1^ Department of Cardiology, The 2nd Affiliated Hospital of Harbin Medical University, Harbin, China; ^2^ State Key Laboratory of Frigid Zone Cardiovascular Diseases (SKLFZCD), Harbin Medical University, Harbin, China; ^3^ The Key Laboratory of Myocardial Ischemia, Chinese Ministry of Education, Harbin, China

**Keywords:** major adverse cardiovascular and cerebrovascular events, optical coherence tomography, plaque vulnerability, ST-segment elevation myocardial infarction, triglyceride-glucose index

## Abstract

**Background:**

The triglyceride-glucose (TyG) index is a reliable surrogate marker for insulin resistance, and is associated with cardiovascular diseases. However, the specific impact of TyG index on coronary plaque vulnerability and long-term outcomes in patients with ST-segment elevation myocardial infarction (STEMI) has not been fully investigated. This study aimed to investigate the association of the TyG index with coronary plaque characteristics and clinical outcomes.

**Methods:**

Between January 2017 to December 2019, 1,831 STEMI patients who underwent optical coherence tomography imaging were retrospectively enrolled. Patients were divided into three groups based on TyG index tertiles (Group T1: <8.82, Group T2: 8.82-9.41, Group T3: ≥9.41). Major adverse cardiovascular and cerebrovascular events (MACCE) included cardiac death, non-fatal stroke, non-fatal myocardial infarction, ischemia-driven revascularization, and rehospitalization.

**Results:**

The average age was 58.7 years, and 72.1% were male. The incidence of plaque rupture, thin-cap fibroatheromas, macrophages, and the size of lipid core, increased with increasing TyG index tertiles (all *P*<0.05). Multivariate logistic regression analysis showed that TyG index independently predicted culprit plaque rupture (T2: OR 1.39, 95%CI 1.06-1.82; T3: OR 1.51, 95%CI 1.05-2.16; T1 as reference). During a median follow-up of 4.2 years, 541 (29.9%) patients developed MACCE. Patients in the highest TyG index tertile had a significantly higher cumulative incidence of MACCE (43.5% vs. 37.3% vs. 31.1%, *P* = 0.007) than the other two groups. After adjusting for clinical risk factors and coronary plaque features, the increased TyG index independently predicted MACCE (HR 1.18, 95%CI 1.00-1.38, per unit increased). This association was notable in patients without diabetes but was not demonstrable in diabetes (interaction *P*-value <0.05).

**Conclusions:**

In patients with STEMI, elevated TyG index increased atherosclerotic plaque vulnerability, and independently predicted plaque rupture. A higher TyG index was an independent predictor of MACCE, especially for patients without diabetes.

## 1 Introduction

Acute myocardial infarction (AMI) is a major cause of death and disability worldwide and is usually triggered by ruptured or eroded atherosclerotic plaque (1, 2). Insulin resistance (IR) is known to be one of the major risk factors for atherosclerosis, and is strongly linked to the occurrence and development of cardiovascular disease (CVD) (3, 4). The triglyceride-glucose (TyG) index, calculated using fasting blood glucose (FBG) and triglyceride (TG) levels, has been proposed as a reliable and applicable surrogate marker of IR. Some studies have shown that elevated TyG index was significantly associated with an increased risk of CVD in healthy individuals (5–7), and an increased incidence of subsequent adverse cardiovascular events in patients with CVD (8–10). However, the exact mechanism underlying this relationship remains unknown.

Previous computed tomography angiography (CTA) studies indicated that elevated TyG index can independently predict functionally coronary stenosis and low-attenuation plaque in hypertensive patients (11), as well as the presence and progression of coronary artery calcification in the general population (12–14). However, another recent CTA study found that diabetic patients with a higher TyG index had more high-risk plaque features but fewer calcified plaques (15). Evidence from two relatively small coronary intravascular imaging studies of patients with acute coronary syndrome (ACS) indicated a potential correlation between high TyG index levels and unstable coronary plaque (16, 17). However, current evidence on the association between the TyG index and coronary plaque vulnerability is limited, particularly among patients with ST-segment elevation myocardial infarction (STEMI). Moreover, few studies have investigated the long-term impact of the TyG index on adverse cardiovascular outcomes in STEMI patients after percutaneous coronary intervention (PCI).

Therefore, this study aimed to evaluate the association between the TyG index and coronary atherosclerotic plaque characteristics, as well as long-term clinical outcomes, in a large cohort of STEMI patients who underwent optical coherence tomography (OCT) imaging.

## 2 Materials and methods

### 2.1 Study design and population

**Figure 1:**
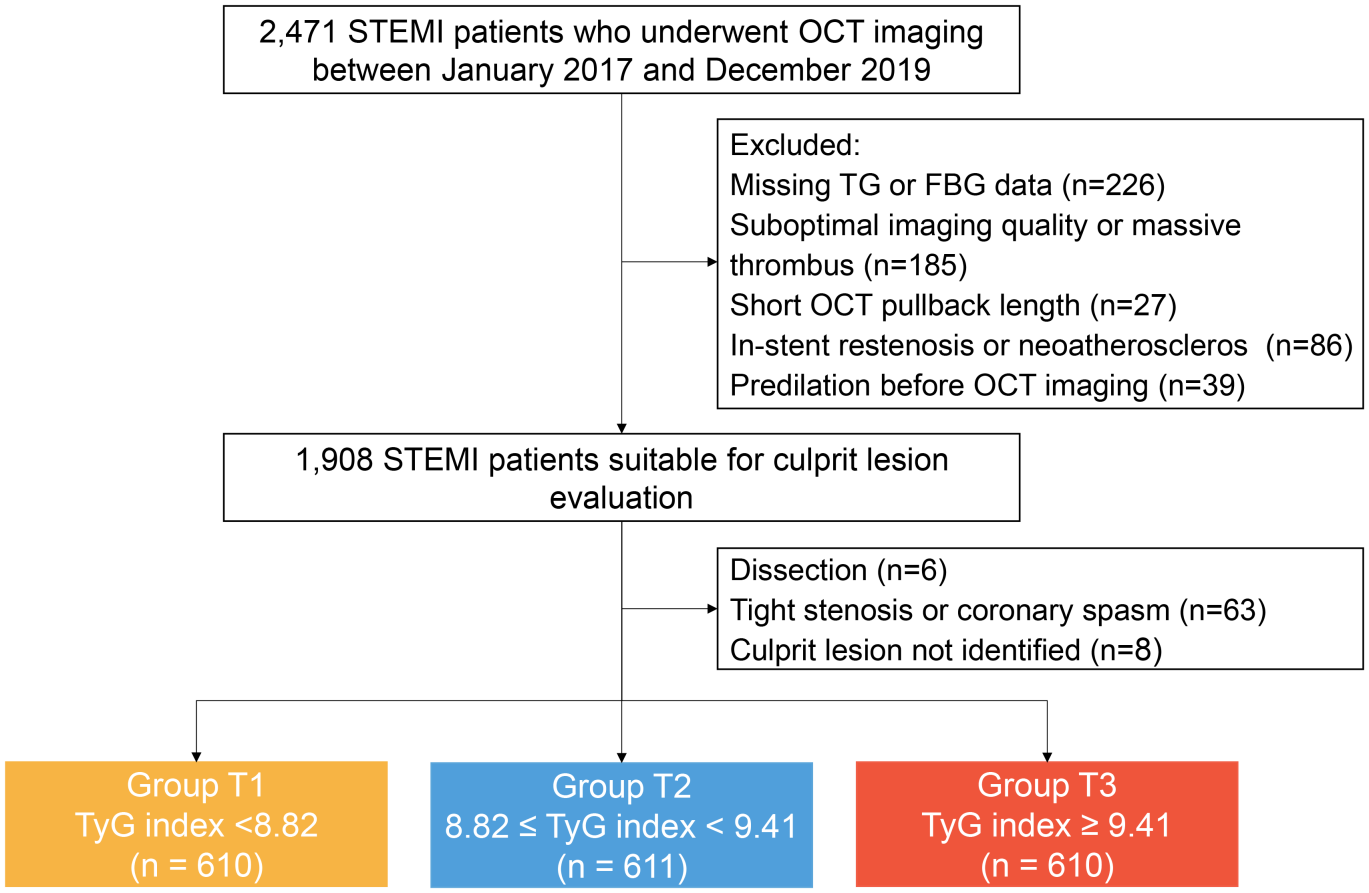
Study flow chart. FBG, fasting blood-glucose; OCT, optical coherence tomography; STEMI, ST-segment elevation myocardial infarction; TyG, triglyceride glucose; TG, triglyceride.

Study participants were retrospectively enrolled at the Second Affiliated Hospital of Harbin Medical University. Patients with STEMI (aged ≥18 years) and undergoing pre-intervention OCT imaging of the culprit lesion during primary PCI were eligible for inclusion. The main exclusion criteria were cardio-genic shock, end-stage renal disease, serious liver dysfunction, and allergy to contrast media. Patients with left main disease, chronic total occlusion, or extremely tortuous or heavily calcified vessels were not included owing to the anticipated difficulties in performing OCT. From January 2017 to December 2019, a total of 2,471 eligible patients with STEMI who underwent pre-intervention OCT imaging were retrospectively selected. There were 640 patients excluded from analysis for the following reasons: incomplete data records of fasting triglyceride (TG) or fasting blood glucose (FBG) at baseline (n=226), suboptimal image quality or massive thrombus (n=185), short OCT pullback length (n=27), in-stent restenosis or neoatherosclerosis (n=86), predilation before OCT imaging (n=39), dissection (n=6), tight stenosis or coronary spasm (n=63), and culprit lesion not identified (n=8). Finally, 1,831 patients with STEMI were included in the analysis. All enrolled patients were divided into three groups across tertiles of the TyG index: Group T1 (TyG index <8.82, n=610), Group T2 (8.82≤ TyG index <9.41, n=611), Group T3 (TyG index ≥9.41, n=610). The study flowchart is displayed in **Figure 1**.

The TyG index was computed using the following formula: Ln [fasting TG (mg/dL) × FBG (mg/dL)/2] (18). Venous blood samples were drawn after a 12-hour overnight fast, and plasma was immediately separated by centrifugation. FBG, total cholesterol (TC), TG, low-density lipoprotein cholesterol (LDL-C), and high-density lipoprotein cholesterol (HDL-C), was measured using a Roche Cobas c702 analyzer (Roche Diagnostics). The clinical diagnosis of STEMI and definitions of traditional coronary risk factors are described in the **Supplementary Data**. The need for written informed consent was waived by the Ethics Committee of the Second Affiliated Hospital of Harbin Medical University due to the retrospective nature of the study. The study was performed in accordance with the Declaration of Helsinki.

### 2.2 Coronary angiography analysis

Quantitative coronary angiography (QCA) analysis was conducted using the Cardiovascular Angiography Analysis System (CAAS version 5.10, Pie Medical Imaging B.V., Maastricht, the Netherlands) by an independent investigator who was blinded to patient clinical information and OCT findings. Coronary flow was assessed using the Thrombolysis in Myocardial Infarction (TIMI) flow grade. End-diastolic frames were selected for analysis, and the tip of the catheter was used for calibration. Lesion location, lesion length, reference vessel diameter, minimal lumen diameter, and percentage diameter stenosis were measured at culprit lesions.

### 2.3 OCT image acquisition and analysis

The OCT imaging was performed using a commercially available frequency-domain OCT system (ILUMIEN OPTIS System, Abbott Vascular, Santa Clara, CA). For patients with thrombolysis in myocardial infarction flow grade <2 and/or occlusive thrombosis, manual thrombectomy was allowed before OCT imaging. The detailed process of OCT imaging has been described previously (19). All OCT images were submitted to the Intravascular Imaging and Physiology Core Lab of the Second Affiliated Hospital of Harbin Medical University and analyzed using off-line review workstation software (Abbott Vascular) by 2 experienced investigators who were blinded to the patients’ information. Any discordance between the 2 investigators was resolved by a consensus from a third investigator. The culprit lesion was identified based on angiographic findings, electrocardiogram changes, or echocardiographic left ventricular wall motion abnormalities (20). In patients with multiple stenoses, the plaque with the most severe stenosis or evidence of acute thrombus on angiography or OCT was considered to be the culprit. Quantitative and qualitative OCT analyses of the culprit lesions were performed according to previously described criteria and consensus (21), and detailed in the **Supplementary Data**.

### 2.4 Clinical outcome

All enrolled patients received scheduled follow-ups at 1, 3, 6, and 12 months and annually thereafter by telephone interview or clinical visit after discharge. Follow-up continued until death, loss to follow-up, or 31 August 2023. Major adverse cardiovascular and cerebrovascular events (MACCE) were defined as the composite of cardiac death, non-fatal stroke, non-fatal myocardial infarction (MI), ischemia-driven revascularization, and rehospitalization for unstable or progressive angina. Detailed definitions of the individual event are provided in the **Supplementary Data**.

### 2.5 Statistical analysis

All statistical analysis was performed using SPSS software (version 26; IBM Corp, Armonk, NY, USA). A two-tailed *P*-value <0.05 indicates that the difference is statistically significant. The nonparametric one-sample Kolmogorov–Smirnov test was used to assess the normality of continuous variables. Normally distributed variables are described as mean ± standard deviation (SD) and compared by using the Analysis of Variance (*ANOVA*), whereas non-normally distributed variables are described as median (interquartile range [IQR]) and compared by using the Kruskal Wallis *H* test. Categorical variables were presented as number (percentage), and compared using either a Chi-square test or Fisher exact test, as appropriate. To take into account the within-subject correlation due to multiple calcified plaques analyzed within the same lesion, comparisons of plaque characteristics between different groups were carried out using the Generalized Estimating Equations approach.

The Pearson (normal distribution) or Spearman (non-normal distribution) correlation test was used to discern relationships between the TyG index and cardiovascular risk factors. The association between the TyG index and lipid length, lipid index, calcium length, and maximum calcium thickness was assessed using the multivariate linear regression analysis. The results were presented as standardized regression coefficients (β), using Group T1 as reference. The association of the TyG index with plaque rupture, TCFA, macrophage, and cholesterol crystals was evaluated by the multivariate logistic regression analysis. The results were presented as odds ratios (OR) with 95% confidence interval (CI), using Group T1 as reference. The Kaplan-Meier method was used to estimate the cumulative event rate, and Log-rank tests were used for comparisons of differences between the groups. Multivariate Cox proportional hazard model was performed to evaluate the association between the TyG index and clinical outcomes. The assumption of proportionality was assessed graphically using a log cumulative hazard plot and by examining the scaled Schoenfeld residuals. Multicollinearity was assessed using variance inflation factors, with all values below 10, suggesting no significant multicollinearity issues. The TyG index was entered into the model as a continuous and categorical variable (tertiles of the TyG index), respectively, and results are reported as hazard ratios (HR) and 95% CI. To assess the incremental predictive performance of outcomes after introducing the TyG index to the baseline risk model, various measures were used including the calculation of the C statistic, continuous net reclassification improvement (NRI), and integrated discrimination improvement (IDI). All the variables that showed *P* values <0.1 in the univariate model were simultaneously entered into the multivariate model.

## 3 Results

### 3.1 Baseline clinical characteristics

A total of 1,831 patients with STEMI were included in the final analysis. The mean age of patients was 58.7 ± 11.6 years, and 1320 (72.1%) were male. The mean TyG index was 9.49 ± 0.73. The baseline clinical characteristics of patients by the TyG index tertiles are summarized in **Table 1**. Patients with the highest TyG index tended to be younger, less likely to be male, and to have higher proportion of diabetes mellitus, hypertension, dyslipidemia, and higher levels of TC, high-sensitive C-reactive protein (hs-CRP), glycated hemoglobin c (HbA1c), in comparison with the other two groups (all *P<*0.001). Details of dyslipidemia summary in **Supplementary Table S1**. Correlation analyses (**Supplementary Table S2**) revealed that the TyG index was positively correlated with TC, TG, LDL-C, FBG, HbA1c, and hs-CRP and negatively correlated with age and HDL-C (all *P*<0.05).

**Table 1:** Baseline clinical characteristics of STEMI patients stratified according to tertile of TyG index.

Variables	Group T1 (n=610)	Group T2 (n=611)	Group T3 (n=610)	*P*-value	P^*^-value		
					T1 vs. T2	T1 vs. T3	T2 vs. T3
Age, years	60.3 ± 11.1	58.2 ± 11.8	57.4 ± 11.6	<0.001	0.001	<0.001	0.253
Male	476 (78.0)	446 (73.0)	398 (65.2)	<0.001	0.048	<0.001	0.004
BMI, kg/m²	24.1 ± 3.8	25.0 ± 3.6	26.0 ± 4.0	<0.001	<0.001	<0.001	<0.001
Current smokers	351 (57.5)	347 (56.8)	305 (50.0)	0.014	0.791	0.008	0.017
Risk factors							
Hypertension	271 (44.4)	297 (48.6)	358 (58.7)	<0.001	0.159	<0.001	0.001
Dyslipidemia	258 (42.3)	393 (64.3)	566 (92.8)	<0.001	<0.001	<0.001	<0.001
Diabetes mellitus	40 (6.6)	134 (21.9)	334 (54.8)	<0.001	<0.001	<0.001	<0.001
CKD	95 (15.6)	99 (16.2)	102 (16.7)	0.862	NA	NA	NA
Previous history							
Previous MI	26 (4.3)	29 (4.7)	33 (5.4)	0.642	NA	NA	NA
Previous stroke	96 (15.7)	110 (18.0)	103 (16.9)	0.572	NA	NA	NA
Previous PCI	7 (1.1)	11 (1.8)	17 (2.8)	0.109	NA	NA	NA
Laboratory data							
TyG index	8.4 ± 0.33	9.10 ± 0.17	9.96 ± 0.46	<0.001	<0.001	<0.001	<0.001
FBG, mmol/L	6.9 ± 1.6	8.4 ± 2.8	11.7 ± 4.7	<0.001	<0.001	<0.001	<0.001
TG, mg/dL	77.1 (61.1-92.1)	127.5 (100.5-155.9)	210.8 (158.8-279.0)	<0.001	<0.001	<0.001	<0.001
TC, mg/dL	172.8 ± 37.4	184.1 ± 40.8	192.5 ± 41.8	<0.001	<0.001	<0.001	<0.001
LDL-C, mg/dL	110.1 ± 33.6	117.2 ± 34.6	113.4 ± 33.4	0.001	<0.001	0.078	0.057
HDL-C, mg/dL	52.9 ± 12.8	49.6 ± 11.4	44.8 ± 10.2	<0.001	<0.001	<0.001	<0.001
HbA1c, %	5.6 ± 0.5	6.1 ± 1.0	7.2 ± 1.8	<0.001	<0.001	<0.001	<0.001
hs-CRP, mg/L	3.9 (1.7-10.2)	4.2 (2.1-10.0)	5.6 (2.5-11.6)	<0.001	0.171	<0.001	0.001
eGFR, ml/min/1.73 m^2^	82.5 (67.4-104.4)	87.6 (67.4-105.9)	84.8 (66.2-104.8)	0.284	NA	NA	NA
Urea nitrogen, μmol/L	5.6 (4.7-6.8)	5.6 (4.5-6.8)	5.6 (4.6-6.7)	0.803	NA	NA	NA
LVEF, %	57.5 ± 7.0	57.5 ± 7.3	57.7 ± 7.0	0.833	NA	NA	NA

Values are n (%), mean ± SD, or median (IQR). A *P*-value <0.05 or *P* ^*^-value <0.017 was considered statistically significant. BMI, body mass index; CKD, chronic kidney disease; eGFR, estimated glomerular filtration rate; FBG, fasting blood-glucose; HbA1c, glycated hemoglobin c; HDL-C, high-density lipoprotein cholesterol; hs-CRP, high-sensitive C-reactive protein; LDL-C, low-density lipoprotein cholesterol; LVEF, left ventricular ejection fraction; MI, myocardial infarction; NA, not applicable; PCI, percutaneous coronary intervention; TC, total cholesterol; TG, triglyceride; TyG index, triglyceride glucose index.

### 3.2 Angiographic findings of culprit lesions

The lesion distribution and QCA data are listed in **Table 2**. The majority of analyzed culprit lesions were located in the left anterior descending artery, 57.5% in Group T1, 48.3% in Group T2, and 43.6% in Group T3 (*P*<0.001). Patients in Group T3 presented more frequently with initial TIMI flow grade ≤1 (68.0% vs. 58.2%, *P* = 0.001) and multivessel disease (77.7% vs. 68.7%, *P*<0.001), compared with those in Group T1. No significant inter-group differences were observed in the QCA findings of culprit lesions (all *P>*0.05).

**Table 2:** Angiographical findings of culprit lesions.

Variables	Group T1 (n=610)	Group T2 (n=611)	Group T3 (n=610)	*P*-value	P^*^-value		
					T1 vs. T2	T1 vs. T3	T2 vs. T3
Culprit lesion vessel				<0.001	0.006	<0.001	0.143
LAD	351 (57.5)	295 (48.3)	266 (43.6)				
LCX	63 (10.3)	70 (11.5)	68 (11.1)				
RCA	196 (32.1)	246 (40.3)	276 (45.2)				
Culprit lesion site				0.219	NA	NA	NA
Proximal segment	250 (41.0)	246 (40.3)	214 (35.1)				
Mid segment	246 (40.3)	231 (37.8)	251 (41.1)				
Distal segment	99 (16.2)	117 (19.1)	129 (21.1)				
Side branches	15 (2.5)	17 (2.8)	16 (2.6)				
Multivessel disease	419 (68.7)	421 (68.9)	474 (77.7)	<0.001	0.985	<0.001	0.001
Initial TIMI flow ≤1	355 (58.2)	396 (64.8)	415 (68.0)	0.001	0.021	<0.001	0.258
QCA data							
RVD, mm	2.9 (2.6-3.3)	3.0 (2.6-3.4)	2.9 (2.6-3.3)	0.682	NA	NA	NA
MLD, mm	0.8 (0.5-1.1)	0.8 (0.5-1.0)	0.7 (0.5-1.0)	0.076	NA	NA	NA
DS, %	74.0 (63.0-81.0)	74.0 (65.0-82.0)	75.0 (66.0-82.2)	0.098	NA	NA	NA
Lesion length, mm	17.0 (12.3-24.8)	17.1 (12.2-25.1)	17.7 (12.2-26.6)	0.730	NA	NA	NA

Values are n (%) or median (IQR). A *P*-value <0.05 or *P* ^*^-value <0.017 was considered statistically significant. DS, diameter stenosis; LAD, left anterior descending artery; LCX, left circumflex artery; MLD, minimal lumen diameter; NA, not applicable; QCA, quantitative coronary angiography; RCA, right coronary artery; RVD, reference vessel diameter; TIMI, initial thrombolysis in myocardial infarction.

### 3.3 OCT findings of culprit lesions

**Figure 2:**
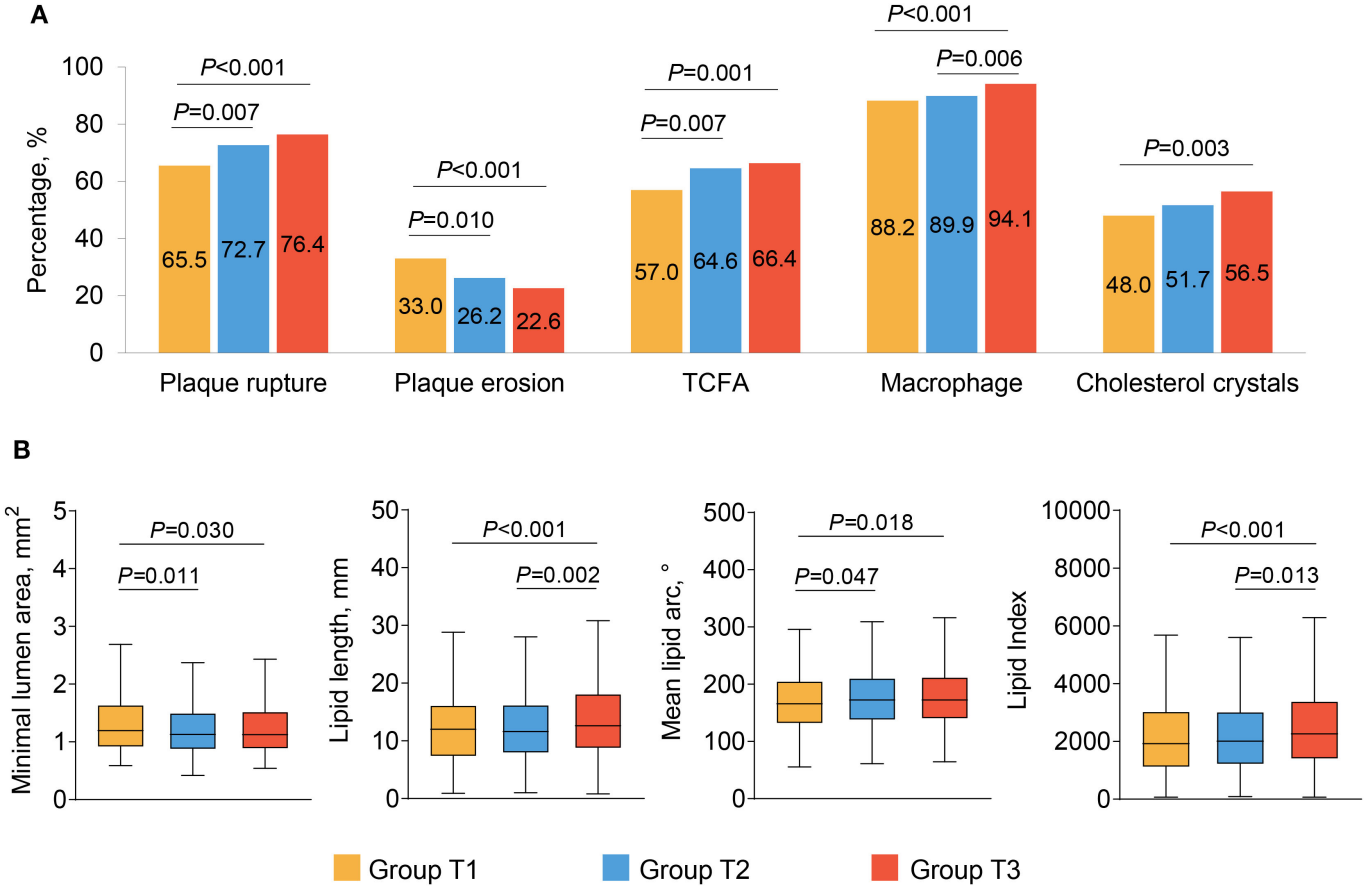
The OCT findings of culprit lesions. Association between the TyG index and OCT qualitative **(A)** and quantitative **(B)** plaque characteristics. OCT, optical coherence tomography; TCFA, thin cap fibroatheroma; TyG, triglyceride glucose.

Comparisons of OCT findings among the three groups are shown in **Figure 2** and **Table 3**. Patients in Group T3 and Group T2 exhibited a higher prevalence of plaque rupture (76.4% vs. 72.7% vs. 65.6%, *P<*0.001) and TCFA (66.4% vs. 64.6% vs. 57.0%, *P* = 0.002), and a lower prevalence of plaque erosion (22.6% vs. 26.2% vs. 33.0%, *P<*0.001) than patients in Group T1. A more frequent incidence of lipid-rich plaque, macrophage, and cholesterol crystals was also observed in patients with the highest TyG index, compared with those with the lowest TyG index (all *P<*0.05). Patients with the highest TyG index had longer lipid length, greater lipid index, and smaller minimal lumen area (*P*<0.05).

**Table 3:** OCT findings of culprit lesions.

Variables	Group T1 (n=610)	Group T2 (n=611)	Group T3 (n=610)	*P*-value	P^*^-value		
					T1 vs. T2	T1 vs. T3	T2 vs. T3
Culprit lesion type							
Plaque rupture	400 (65.6)	444 (72.7)	466 (76.4)	<0.001	0.007	<0.001	0.135
Plaque erosion	201 (33.0)	160 (26.2)	138 (22.6)	<0.001	0.010	<0.001	0.147
Calcified nodule	9 (1.5)	6 (1.0)	6 (1.0)	0.647	NA	NA	NA
Mean RLA, mm^2^	7.2 (5.6-9.2)	7.3 (5.5-9.3)	7.0 (5.4-9.3)	0.771	NA	NA	NA
Lesion length, mm	18.0 (14.0-24.0)	18.8 (14.6-24.0)	19.0 (14.6-25.0)	0.223	NA	NA	NA
MLA, mm^2^	1.2 (0.9-1.6)	1.1 (0.9-1.5)	1.1 (0.9-1.5)	0.023	0.011	0.030	0.728
AS, %	82.5 (76.8-87.1)	83.7 (78.2-87.8)	83.4 (76.2-87.6)	0.052	NA	NA	NA
Lipid plaque	542 (88.9)	558 (91.3)	575 (94.3)	0.003	0.148	0.001	0.047
Lipid length, mm	10.8 (6.0-15.4)	11.0 (7.0-15.8)	12.0 (8.0-17.6)	<0.001	0.224	<0.001	0.002
Mean lipid arc, °	165.9 (132.8-203.8)	172.4 (138.8-209.6)	172.4 (141.3-210.7)	0.040	0.047	0.018	0.659
Maximum lipid arc, °	311.8 (221.4-360.0)	324.4 (234.9-360.0)	360.0 (238.8-360.0)	0.120	NA	NA	NA
Lipid index	1920.7 (1128.6-3010.1)	2009.0 (1232.0-2993.4)	2259.7 (1418.6-3369.5)	0.001	0.172	<0.001	0.013
Minimal FCT, μm	60.0 (46.7-77.0)	57.0 (43.0-70.0)	57.0 (46.7-70.0)	0.263	NA	NA	NA
Lipid-rich plaque	539 (88.4)	556 (91.0)	572 (93.8)	0.004	0.130	0.001	0.068
TCFA	348 (57.0)	395 (64.6)	405 (66.4)	0.002	0.007	0.001	0.521
Macrophage	538 (88.2)	549 (89.9)	573 (94.1)	0.001	0.355	<0.001	0.006
Microvessel	243 (39.8)	235 (38.5)	249 (41.0)	0.659	NA	NA	NA
Cholesterol crystals	293 (48.0)	316 (51.7)	344 (56.5)	0.012	0.198	0.003	0.095
Thrombus	592 (97.0)	589 (96.4)	590 (96.7)	0.816	NA	NA	NA
Calcification	272 (44.6)	252 (41.2)	260 (42.6)	0.494	NA	NA	NA
Spotty calcification	188 (30.8)	185 (30.3)	199 (32.6)	0.652	NA	NA	NA
Superficial-65 calcification	187 (30.7)	147 (24.1)	147 (24.1)	0.011	0.010	0.010	0.987
Superficial-100 calcification	221 (36.2)	180 (29.5)	174 (28.5)	0.007	0.012	0.004	0.719

Values are n (%) or median (IQR). A *P*-value <0.05 or *P* ^*^-value <0.017 was considered statistically significant. AS, area stenosis; FCT, fibrous cap thickness; MLA, minimal lumen area; NA, not applicable; OCT, optical coherence tomography; RLA, reference lumen area; TCFA, thin cap fibroatheroma.

Among all enrolled patients, 42.8% had coronary calcification, and patients in Group T1 had highest prevalence of superficial-65 calcification (30.7% vs. 24.1% vs. 24.1%, *P* = 0.011) and superficial-100 calcification (36.2% vs. 29.5% vs. 28.5%, *P* = 0.007) than those in Group T2 and T3 (**Table 3**). The quantitative OCT analyses of all coronary calcifications are displayed **Table 4**; **Supplementary Table S3**. The lesion-level analyses (**Table 4**) showed that lesions in Group T1 had longer, thicker, more superficial calcifications, which were also large in size, as compared with those in Group T2 and T3. Similar results were found in single calcium-level analyses (**Supplementary Table S3**).

**Table 4:** OCT analysis of calcification (lesion level).

Variables	Group T1 (n=272)	Group T2 (n=252)	Group T3 (n=260)	*P*-value	P^*^-value		
					T1 vs. T2	T1 vs. T3	T2 vs. T3
Total calcification number	2.0 (1.0-3.0)	2.0 (1.0-3.0)	2.0 (1.0-3.0)	0.434	NA	NA	NA
Calcium length, mm	6.9 (3.4-12.1)	5.4 (2.6-9.0)	5.5 (2.8-9.8)	0.010	0.004	0.036	0.364
Mean calcium arc, °	70.9 (49.6-95.0)	62.1 (43.4-92.2)	68.2 (45.1-94.0)	0.139	NA	NA	NA
Maximal calcium arc, °	110.5 (75.2-165.0)	99.6 (62.4-172.4)	107.9 (65.9-175.5)	0.269	NA	NA	NA
Calcium index	498.4 (171.0-1120.7)	351.7 (132.0-706.2)	355.4 (154.4-892.6)	0.016	0.005	0.080	0.289
Maximal calcium thickness, µm	965.0 (740.0-1220.0)	855.0 (580.0-1130.0)	850.0 (620.0-1120.0)	<0.001	<0.001	0.001	0.678
Mean calcium depth, µm	171.0 (120.0-239.0)	187.0 (124.8-280.0)	191.0 (123.0-285.0)	0.140	NA	NA	NA
Minimal calcium depth, µm	40.0 (20.0-80.0)	50.0 (23.0-127.8)	40.0 (20.0-103.0)	0.033	0.009	0.481	0.100
Mean calcium area, mm^2^	1.0 (0.5-1.6)	0.8 (0.4-1.3)	0.8 (0.4-1.4)	0.005	0.002	0.023	0.373
Maximal calcium area, mm^2^	2.0 (1.1-3.5)	1.5 (0.7-3.0)	1.7 (0.8-3.2)	0.006	0.002	0.044	0.284

Values are median (IQR). A *P*-value <0.05 or *P* ^*^-value <0.017 was considered statistically significant. NA, not applicable; OCT, optical coherence tomography.

Multivariable regression analyses (**Supplementary Table S4**) revealed a significant correlation between the TyG index and culprit plaque rupture. The risk of plaque rupture was 1.39 (95%CI: 1.06-1.82) and 1.51 (95%CI: 1.05-2.16) times increased among patients in Group T2 and Group T3, respectively, as compared to those in Group T1. Although the TyG index correlated with TCFA, macrophage, cholesterol crystals, lipid length, lipid index, calcium length, and maximum calcium thickness in univariable analysis, the TyG index did not serve as an independent clinical predictor for the features of above-vulnerable plaque in the multivariable analysis.

### 3.4 Clinical outcomes

**Figure 3:**
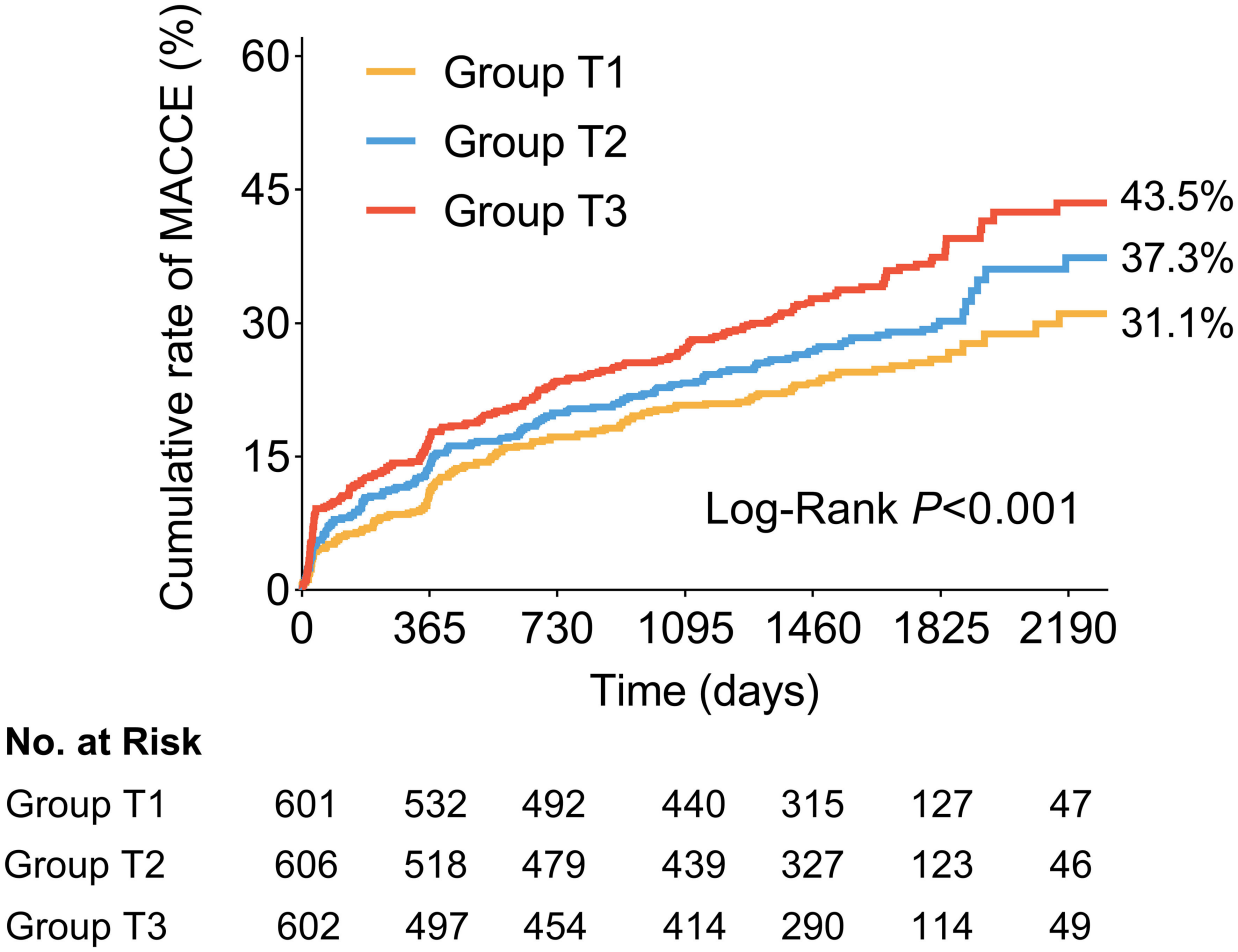
Kaplan-Meier time-to-first event curves for MACCE. Patients in the highest TyG index tertile had a significantly greater incidence of MACCE. MACCE was defined as composite of cardiac death, non-fatal stroke, non-fatal myocardial infarction, ischemia-driven revascularization, and rehospitalization for unstable or progressive angina. MACCE, major adverse cardiovascular and cerebrovascular events; TyG, triglyceride glucose.

The clinical follow-up data were available in 98.8% (1809/1831) of patients. During a median follow-up of 4.2 years (IQR: 4.0-5.0 years), 538 patients (29.7%) experienced MACCE after discharge, including 91 (5.0%) cardiac death, 87 (4.8%) non-fatal stroke, 61 (3.4%) non-fatal MI, 263 (14.5%) ischemia-driven revascularization, and 221 (12.2%) rehospitalization (**Table 5**). As shown in **Figure 3**, the cumulative incidence of MACCE increased with increasing tertiles of the TyG index, with the highest incidence in Group T3 (31.1% vs. 37.3% vs. 43.5%, Log-rank *P<*0.001). There were no significant differences in discharge medication among the three groups (**Supplementary Table S5**).

**Table 5:** Clinical outcome.

Events	Group T1 (n=601)	Group T2 (n=606)	Group T3 (n=602)	Log-Rank *P* value
MACCE	150 (31.1)	175 (37.3)	213 (43.5)	<0.001
Cardiac death	21 (5.1)	33 (6.0)	37 (6.7)	0.096
Non-fatal stroke	19 (4.4)	27 (6.3)	41 (7.9)	0.011
Non-fatal MI	21 (4.2)	16 (4.6)	24 (5.1)	0.433
Revascularization	84 (15.3)	86 (18.7)	93 (19.0)	0.633
Rehospitalization for unstable or progressive angina	63 (16.9)	72 (16.0)	86 (23.5)	0.135
All-cause death	43 (10.0)	54 (11.9)	69 (14.6)	0.040

Values are n (%). Percentages and *P*-value are from Kaplan-Meier estimated cumulative rate and Log-rank test, respectively. MACCE was defined as composite of cardiac death, non-fatal stroke, non-fatal MI, ischemia-driven revascularization, and rehospitalization for unstable or progressive angina. MI, myocardial infarction; MACCE, major adverse cardiovascular and cerebrovascular events.

The univariate and multivariate Cox proportional hazard models to predict MACCE are shown in **Supplementary Table S6**; **Table 6**. After adjusting for clinical and medications and OCT features, whether analyzed as a continuous variable or a categorical variable, the TyG index was independently associated with a higher risk of MACCE. As compared with patients in Group T1, the fully adjusted HR for MACCE was 1.18 (95%CI: 0.93-1.50) and 1.37 (95%CI: 1.05-1.79) in patients with Group T2 and Group T3, respectively. The increased risk of MACCE from Group T1 to Group T3 was statistically significant (*P* = 0.048). In addition, for the per unit increase in the TyG index, the risk of incident MACCE increased by 18% (HR: 1.18, 95%CI: 1.00-1.38) among the fully adjusted model. A 12% increased risk of MACCE was observed per SD increase in the TyG index (HR: 1.12, 95%CI: 1.00-1.26). The addition of the TyG index into the baseline risk model, which contained age, gender, hypertension, dyslipidemia, diabetes mellitus, previous myocardial infarction, TC, LDL-C, HDL, HbA1c, hs-CRP, improved the prediction probability for MACCE (C-statistic *P* = 0.038; NRI [95%CI]=0.230 [0.132-0.340], *P*<0.001; IDI [95%CI]=0.008 [0.004-0.012], *P*<0.001; **Supplementary Table S7**).

**Table 6:** Univariate and multivariate Cox regression analysis for MACCE.

Variables	HR (95%CI)		
	Model 1	Model 2	Model 3
TyG index as a continuous variable			
Per 1 unit increase	1.23 (1.10-1.38) ^**^	1.19 (1.01-1.39) ^*^	1.18 (1.00-1.38) ^*^
Per 1 SD increase	1.16 (1.07-1.26) ^**^	1.13 (1.01-1.27) ^*^	1.12 (1.00-1.26) ^*^
TyG index as a nominal variable			
Group T1	1 (Reference)	1 (Reference)	1 (Reference)
Group T2	1.19 (0.96-1.48)	1.17 (0.93-1.48)	1.18 (0.93-1.50)
Group T3	1.51 (1.22-1.86) ^*^	1.39 (1.06-1.82) ^*^	1.37 (1.05-1.79) ^*^
P for trend	<0.001	0.036	0.048

Values are HR (95%CI). ^*^ *P <*0.05 and ^**^ *P <*0.001. Model 1 unadjusted; Model 2 adjusted for age, sex, BMI, current smokers, hypertension, dyslipidemia, diabetes mellitus, CKD, previous myocardial infarction, TC, LDL-C, HDL-C, HbA1c, hs-CRP, aspirin, P2Y12 inhibitor, statins, β-blockers, ACEI/ARB; Model 3 adjusted for all factors in model 2 plus culprit lesion vessel, multivessel disease, plaque rupture, TCFA, macrophage, microvessels, cholesterol crystals, thrombus, and calcification. ACEI, angiotensin-converting enzyme inhibitor; ARB, angiotensin receptor blocker; CI, confidence interval; HR, hazards ratio; TCFA, thin cap fibroatheroma. Other abbreviations as shown in **Tables 1**, **5**.

Sensitivity analysis was performed to assess the robustness of our findings after excluding patients who had received prior lipid- or glucose-lowering therapy (**Supplementary Tables S7-10**). The TyG index remained an independent predictor of plaque rupture and MACCE, even after adjustment for HbA1c, hs-CRP, and lipid profiles.

### 3.5 Plaque features and clinical outcomes stratified by diabetes mellitus

The incidence of plaque rupture, lipid-rich plaque, and TCFA, as well as the size of lipid core, increased with increasing TyG index in patients without diabetes mellitus (**Supplementary Table S11**). Multivariate logistic regression revealed a 2.12-fold (95%CI: 1.37-3.27) and 1.31-fold (95% CI: 0.98-1.76) increase in the likelihood of coronary plaque rupture in Group T3 and Group T2 than in Group T1 (**Supplementary Table S12**). In contrast, in patients with diabetes mellitus, these vulnerability features were all higher in three groups, without any statistically significant between-group differences (**Supplementary Table S11**). Similarly, the TyG index was independently associated with the occurrence of MACCE in the non-diabetes subgroup (HR: 1.35, 95%CI: 1.12-1.62, per 1 unit increase; **Supplementary Table S13**) but not in the diabetes subgroup (**Supplementary Figure S1**, *P* value for interaction =0.022).

## 4 Discussion

This large observational study of 1,831 patients with STEMI who underwent OCT imaging provides new information on the relationship between serum TyG index and coronary plaque characteristics and long-term prognosis. The main findings of the present study were as follows. First, patients with high TyG index presented with more frequent vulnerable plaque characteristics, such as plaque rupture, TCFA, macrophages, and cholesterol crystals. The TyG index was an independent predictor of plaque rupture. Second, patients with the lowest tertile TyG index had a higher prevalence of superficial calcification and showed more extensive calcification. Third, elevated TyG index was independently associated with an increased risk of MACCE. Fourth, the effect of increased TyG index on plaque rupture and poor prognosis was more pronounced in patients without diabetes. These findings further suggest that the TyG index may be a promising predictor for assessing high-risk lesions and poor prognosis in STEMI patients, thus facilitating risk stratification and management.

### 4.1 TyG index and coronary plaque vulnerability

IR is considered an important risk factor for ASCVD and is associated with worse clinical outcomes (3, 4). The hyperinsulinemic-euglycemic clamp technique is the gold standard for the identification of IR (22), but its routine application in clinical practice is limited by its invasiveness, complexity, and expense. The TyG index is thought to be a reliable biomarker of IR that can comprehensively reflect blood lipid and blood glucose data (18, 23). The homeostatic model assessment of insulin resistance (HOMA-IR) is also a widely adopted surrogate for IR. Previous studies indicated that the TyG index may offer superior discriminative capacity for identifying IR compared with HOMA-IR (24–26). IR disrupts lipid and glucose homeostasis and elevates circulating free fatty acids (FFAs). Excess FFAs can initiate an inflammatory cascade by activating pathways such as the Toll-like receptor 4 and the NOD-like receptor protein 3 inflammasome (27). Concurrently, these metabolic disturbances promote the overproduction of reactive oxygen species, leading to oxidative stress (28). Furthermore, IR selectively impairs PI3K/Akt signalling in endothelial cells, down-regulating endothelial nitric oxide synthase activity and reducing bioavailable nitric oxide, thereby precipitating endothelial dysfunction (3, 27). These processes promote monocyte adhesion and transmigration, foam-cell formation, and smooth-muscle-cell proliferation, ultimately accelerating the initiation and progression of atherosclerotic plaques and increasing their vulnerability.

Recent studies have attempted to link the TyG index to plaque vulnerability. The TyG index has been reported to be independently associated with the number and severity of coronary artery stenoses (29, 30). The PARADIGM sub-study found that baseline total plaque volume and necrotic core volume assessed by CTA increased with increasing TyG index (31). Another CTA study showed a significant correlation between the TyG index and positive remodeling and low-attenuation plaque in hypertensive patients (11). According to an intravascular ultrasound research involving 234 ACS patients, coronary plaques in patients with a higher TyG index were often accompanied by a minimum lumen area ≤4.0mm² or a plaque burden >70% (16). In an OCT study of 110 ACS patients, elevated TyG index independently predicted the occurrence of plaque rupture and TCFA in the coronary nonculprit region (17). In contrast, an OCT study by Zhao et al., which included 274 STEMI patients, reported that the TyG index was not associated with AS plaque morphology. Still, a significant association between the TyG index and cardiac events was found only among patients with plaque rupture (32).

In the present study, based on the larger cohort of patients with STEMI undergoing OCT imaging, we found that a higher TyG index was associated with a more typically vulnerable plaque phenotype, manifested by more lipid plaques, TCFA, plaque rupture incidence, and larger lipid cores. In addition, we discovered that the elevated TyG index was an independent predictor of culprit plaque rupture. Intravascular imaging studies have shown that plaque rupture is the most common cause of thrombosis and AMI (approximately 70%) (33), most of which have a specific phenotype, TCFA, characterized by a thin fibrous cap covering a large necrotic lipid core with extensive macrophage accumulation (34). Activation of systemic and local inflammation is a key pathogenic mechanism for coronary plaque instability (35). Edgar et al. found that hyperglycaemia promotes monocyte adhesion to endothelial cells and accelerates the inflammatory activity of macrophages (36). Previous CTA studies identified the TyG index level was independently and positively correlated with the pericoronary fat attenuation index, a biomarker of coronary inflammation (11, 37). In this study, the incidence of macrophage infiltration and hsCRP levels increased with increasing TyG index, which supports the idea that the TyG index is closely related to chronic inflammation. As such, TyG index may be used as a potential biomarker for coronary plaque vulnerability in risk stratification in STEMI patients.

### 4.2 TyG index and calcification

Some CTA studies have shown that a higher TyG index was an independent predictor of the presence and progression of coronary calcification in the general population (12–14). In contrast, a recent CTA study found that patients with diabetes mellitus and a higher TyG index had fewer calcified plaques (15). In the current study, no significant difference in the incidence of calcification among the three groups was observed, which aligns with observations from previous intravascular imaging studies (16, 17, 32). The discrepancy in results may be explained by the different study populations. Of note, this study is the first to investigate the association between TyG index and quantitative parameters of calcification by OCT, finding that lesions with the lowest TyG index exhibited a wider range of calcification accumulation and a thinner calcium depth. Krishnamoorthy et al. discovered that coronary calcification volume was inversely related to lipid volume index and mean calcium depth after adjusting for other OCT characteristics (38). Patients with diabetes also had lower coronary calcification volumes than those without. An IVUS study revealed that patients with SAP had a larger area of calcium deposits at the culprit lesion, compared to patients with UAP and AMI (39). Large, dense calcifications may be linked to plaque stability, whereas small calcium deposits, particularly those enclosed in a thin fibrous cap, are more likely to result in unstable plaques (40). This may explain why patients with the lowest TyG index had the largest calcification content.

### 4.3 TyG index and clinical outcome

Growing evidence suggested that an elevated TyG index was related to adverse clinical events. In the general population, an elevated TyG index is significantly associated with an increased risk of cardiovascular disease (5–7). In patients with cardiovascular disease at baseline, an elevated TyG index is a valid predictor of recurrent cardiovascular events (8–10). Luo et al. found that a higher TyG index was an independent predictor of adverse cardiovascular outcomes in STEMI patients within 1 year after PCI (41). But its long-term effects remain unknown. Our results further demonstrated that STEMI patients with a higher TyG index had a significantly greater incidence of 6-year MACCE, and the predictive value was independent of conventional risk factors and OCT features. Furthermore, the addition of the TyG index to the baseline risk model improved its predictive value. Thus, the TyG index may serve as a useful predictor of adverse outcomes in STEMI patients undergoing PCI. Prior studies have demonstrated that the addition of TyG index to existing risk models (e.g., GRACE) had an incremental effect on the predictive value for adverse cardiovascular events (42–45). Future study is needed to investigate whether combining the TyG index with existing risk models improves STEMI risk prediction.

Notably, the correlation between the TyG index and MACCE was significant only in patients without diabetes mellitus, likely due to its high correlation with vulnerable plaque. In contrast, no statistical association was found between the TyG index and adverse events in patients with diabetes mellitus. Several factors may explain this observation. In STEMI patients with diabetes, widespread endothelial dysfunction, vascular calcification, and diffuse atherosclerosis blunt the discriminatory power of the TyG index as a metabolic biomarker (46–48); here, integrating circulating markers with plaque phenotypes may provide superior prognostic value. A study by Kedhi et al. demonstrated that TCFA robustly predicted adverse cardiovascular events in ACS patients with diabetes, whereas no such association was evident in non-diabetic subgroup (49). Moreover, diabetic individuals generally exhibit more severe baseline insulin resistance, resulting in higher TyG values with limited variability, which compromises the index’s capacity to stratify risk and reduces its predictive utility. Concurrent comorbidities - hypertension, dyslipidemia, and chronic inflammation - may further confound the TyG–outcome relationship in this cohort (50). Conversely, among non-diabetic individuals, the TyG index may more effectively identify high-risk patients with underlying metabolic abnormalities who have not yet developed diabetes, thus enhancing its predictive relevance. Previous studies also showed that elevated TyG index has a more significant predictive value for prognosis in individuals lacking traditional cardiovascular risk factors, particularly in non-diabetic subgroups (10, 29, 51, 52). Liu et al. found that TyG index can be used to identify individuals at risk of developing CVD in the non-diabetic population cohort (6). These findings underscore the importance of monitoring the TyG index to identify high-risk patients in the non-diabetic subgroup. However, further large prospective studies are needed to evaluate whether lowering the TyG index can reduce coronary plaque vulnerability and improve long-term clinical outcomes.

### 4.4 Clinical implication

Our study found that patients with higher TyG index had higher levels of coronary culprit plaque vulnerability, and elevated TyG index was independently related to plaque rupture. In addition, a higher TyG index was an independent predictor of major adverse cardiovascular events in STEMI patients. These data indicated that a higher level of TyG index represents high-risk plaques and high-risk patients to a certain extent. Importantly, the TyG index also has the advantage of being readily available in a clinical setting, and has been shown to be highly correlated with IR. Notably, the TyG index represents a valuable and cost-effective tool for refining cardiovascular risk assessment in non-diabetic populations, whereas its utility in diabetic patients is limited. Future studies should prioritize the development of integrated risk-stratification tools tailored to diabetics, incorporating multimodal data such as clinical parameters, advanced biomarkers, and high-risk plaque features.

### 4.5 Study limitations

Our study also has several limitations. First, this was a single-center, retrospective, observational study, selection bias was inevitable. Second, in STEMI patients undergoing PCI, the decision to perform OCT was left to the operator’s discretion, thus results must be interpreted with caution as the sample was not representative of all STEMI patients. Third, all patients were enrolled in China. So, our findings might not be generalizable to patients in other countries. Fourth, although we adjusted for potential confounding factors, residual confounding from unknown or unmeasured factors may still exist. Fifth, only the baseline TyG index was included, and the change in the TyG index during the follow-up period was not considered, which may impact the prognosis. Sixfth, thrombus aspiration prior to OCT imaging to restore blood flow may alter lesion plaque characteristics. Seventh, the exact measurements of necrotic core, plaque burden, and some deep micro-structures by OCT are not possible because of the relatively shallow axial penetration. Finally, we did not compare the TyG index with the hyperinsulinemic-euglycemic clamp test and HOMA-IR.

## 5 Conclusion

Patients with higher TyG index levels had increased plaque vulnerability and were more likely to have plaque rupture. Elevated TyG index level was a strong independent predictor of an increased risk of MACCE in STEMI patients, especially among those without diabetes.

## Data Availability

The original contributions presented in the study are included in the article/**Supplementary Material**. Further inquiries can be directed to the corresponding authors.
