# Supplementary material for: The triglyceride-glucose index is associated with coronary plaque features and clinical outcomes in patients with ST-segment elevation myocardial infarction
Source: Front Endocrinol (Lausanne). 2025 Sep 17;16:1665292. doi: 10.3389/fendo.2025.1665292 (PMC12483882; doi:10.3389/fendo.2025.1665292)
Supplement: Supplementary file 1 [file DataSheet1.pdf]

Supplementary Material

1 Supplementary Data

1.1 The clinical diagnosis of ST-segment elevation myocardial infarction (STEMI)

STEMI was defined as continuous chest pain that lasted >30 minutes, arrival at the hospital within 12 hours from the onset of symptoms/symptom onset, ST-segment elevation >0.1 mV in at least 2 contiguous leads, or new-onset left bundle-branch block on the 12-lead electrocardiogram (ECG), and elevated of cardiac markers (creatine kinase-myocardial band or troponin T/I).[1]

1.2 Definition of traditional coronary risk factors

Patients with active smoking within 1 month and no smoking for >1 month were defined as current smokers and former smokers, respectively. Diabetes mellitus was diagnosed if a patient met 1 of the following criteria: documented history or self-reported clinician diabetes mellitus, taking hypoglycemic medicine, fasting glucose ≥126mg/dL, 2h plasma glucose level ≥200mg/dL, classic symptom with casual plasma glucose level ≥200mg/dL, or hemoglobin A1c (HbA1c) ≥6.5%.[2] Hypertension was diagnosed as systolic blood pressure ≥140mmHg or diastolic blood pressure ≥90mmHg or current use of anti-hypertensive agents. Dyslipidemia was defined as total cholesterol (TC) level ≥220mg/dL, triglycerides ≥150 mg/dL, low-density lipoprotein cholesterol (LDL-C) ≥140mg/dL, high-density lipoprotein cholesterol (HDL-C) ≤40mg/dL, or current use of agents for dyslipidemia.[3] The estimated glomerular filtration rate (eGFR) was calculated according to the 2009 Chronic Kidney Disease Epidemiology Collaboration (CKD-EPI) equation. Chronic kidney disease (CKD) was diagnosed/defined as eGFR <60mL/min/1.73m2 for ≥3 months.[4] Body mass index (BMI) was calculated as weight (kg)/[height (m)]^2.

1.3 Quantitative and qualitative analyses of culprit lesion features

Proximal or distal reference was defined as the site with the largest lumen area either proximal or distal to the stenosis, but within the same segment. The average reference lumen (RLA) area was defined as the average of the largest lumen area at the proximal and distal. Lesion length was determined as the distance between the distal and proximal reference. The minimal lumen area (MLA) was the minimal value of the lumen area along the culprit lesion. Percentage lumen area stenosis (AS%) was calculated as (1-MLA / mean RLA) * 100%. Plaque rupture was identified by the presence of fibrous cap discontinuity with a clear cavity formation inside the plaque. Plaque erosion was identified by the presence of attached thrombus overlying an intact fibrous cap, or luminal surface irregularity at the culprit lesion in the absence of thrombus, or attenuation of underlying plaque by thrombus without superficial lipid or calcification immediately proximal or distal to the site of thrombus. Calcified nodule was defined when fibrous cap disruption was detected over a calcified

plaque characterized by protruding calcification into the lumen, superficial calcium, and the presence of substantive calcium proximal and/or distal to the lesion.[5] Plaques were classified into two categories: fibrous plaque was defined by a homogeneous OCT signal with high backscattering; lipid plaque was defined as a signal-poor region with a diffuse border and overlying signal-rich band. For lipid plaque, the lipid length was measured on the longitudinal view. The lipid arc was measured on the cross-sectional view at every 1 mm interval throughout the entire lesion length, and the values were averaged. Lipid index was calculated by multiplication of lipid length and the mean value of lipid arc. Minimal fibrous cap thickness (FCT) was measured three times in the thinnest region of the fibrous cap overlying the lipid pool and the average value was calculated. Lipid-rich plaque (LRP) was defined as a plaque with a maximal lipid arc greater than 90°. TCFA was defined as a plaque with a maximum lipid arc >90° and thinnest FCT <65 μm. Calcification was defined as signal-poor or heterogeneous regions delimited by sharp borders. The calcification arc and calcification depth were evaluated in each cross-sectional view, and the average values were calculated, respectively. Measuring the length of the calcification in the longitudinal view. The calcification index was defined as the product of the average calcification arc and the calcification length. Minimal calcification depth was defined as the minimum distance from the lumen to the superficial calcification edge. There are three types of calcifications: (1) microcalcification was defined as maximal calcium length <1 mm and maximal calcium arc <22.5°, (2) spotty calcification was defined as calcium length ranging from 1 mm to 4 mm or maximal calcium arc ranging from 22.5° to 90°, (3) macrocalcification was defined as maximal calcium length >4 mm or maximal calcium arc >90°.[6] Because of lack of established OCT criteria for superficial calcification, we used calcification depth thresholds of 65 μm and 100 μm to define calcifications as superficial-65 and superficial-100, respectively.[7] Macrophage accumulation was defined as signal-rich, distinct or confluent punctuate regions with heterogeneous backward shadows. Microvessels were defined as a small black hole within a plaque with a diameter of 50-300 μm, signal-poor and sharp-edged, that can usually be recognized on at least 3 consecutive cross-sectional frames. Cholesterol crystals were defined as thin, linear, and high backscattering structures within the plaque. Thrombus was defined as an irregular mass (diameter >250 μm) adherent to the luminal surface or floating within the lumen. The inter-observer agreement for plaque rupture, TCFA, calcification, macrophage, and cholesterol crystals was 0.91, 0.88, 0.90, 0.82, and 0.81, respectively. The intra-observer agreement for these plaque features was 0.93, 0.92, 0.93, 0.87, and 0.83, respectively.

1.4 Definitions of major adverse cardiovascular and cerebrovascular events

All-cause death was defined as death from any cause occurring during follow-up. Cardiac death was defined as death from myocardial infarction, cardiac perforation or pericardial tamponade, arrhythmia or conduction abnormalities, procedural complications, or any death in which a cardiac cause could not be excluded. Non-fatal myocardial infarction was diagnosed by the detection of raise and fall of cardiac biomarkers (preferably troponin) above the 99th centile of the upper reference limit, together with evidence of myocardial ischemia with at least one of the following criteria: ischemic symptoms; ECG changes indicative of

new ischemia (new ST-T changes or new left bundle branch block); development of pathological Q waves in the ECG; and imaging evidence of new loss of viable myocardium or new regional wall motion abnormalities. Ischemia-driven revascularization was defined as either repeat percutaneous or surgical revascularization with ischemic symptoms for the target lesion identified at index procedure or non-target lesions. Scheduled revascularization for lesions that were identified in the index coronary angiograms was not considered as an adverse event. Stroke was defined as a new acute episode of neurologic dysfunction thought to be vascular in origin, with signs or symptoms lasting more than 24 hours, preferably supported by an imaging procedure such as a computed tomography or cardiac magnetic resonance. Rehospitalization for unstable or progressive angina was defined as unscheduled hospitalization for the management of unstable angina or, occurring within 24 hours of the most recent symptoms. Hospitalization was defined as an admission to an inpatient unit or a visit to an emergency department that results in at least a 24-hour stay. Unstable angina was defined as ischemic chest pain (or equivalent) at rest was considered to be myocardial infarction upon final diagnosis and without elevation in cardiac biomarkers of necrosis. Progressive angina was defined as angina class (or equivalent) increases compared to the most recent stable period considered caused by myocardial ischemia based on ECG changes, noninvasive testing, FFR/iFR, not meeting the criteria for myocardial infarction or unstable angina.

References

1 Ibanez B, James S, Agewall S, Antunes MJ, Bucciarelli-Ducci C, Bueno H, et al. 2017 ESC Guidelines for the management of acute myocardial infarction in patients presenting with ST-segment elevation: The Task Force for the management of acute myocardial infarction in patients presenting with ST-segment elevation of the European Society of Cardiology (ESC). *Eur Heart J.* (2018) 39(2): 119-77. doi: 10.1093/eurheartj/ehx393

2 American Diabetes A. Diagnosis and classification of diabetes mellitus. *Diabetes Care.* (2013) 36 Suppl 1(Suppl 1): S67-74. doi: 10.2337/dc13-S067

3 Dai J, Xing L, Jia H, Zhu Y, Zhang S, Hu S, et al. In vivo predictors of plaque erosion in patients with ST-segment elevation myocardial infarction: a clinical, angiographical, and intravascular optical coherence tomography study. *Eur Heart J.* (2018) 39(22): 2077-85. doi: 10.1093/eurheartj/ehy101

4 Levey AS, Stevens LA, Schmid CH, Zhang YL, Castro AF, 3rd, Feldman HI, et al. A new equation to estimate glomerular filtration rate. *Ann Intern Med.* (2009) 150(9): 604-12. doi: 10.7326/0003-4819-150-9-200905050-00006

5 Jia H, Abtahian F, Aguirre AD, Lee S, Chia S, Lowe H, et al. In vivo diagnosis of plaque erosion and calcified nodule in patients with acute coronary syndrome by intravascular optical coherence tomography. *J Am Coll Cardiol.* (2013) 62(19): 1748-58. doi: 10.1016/j.jacc.2013.05.071

6 Milzi A, Burgmaier M, Burgmaier K, Hellmich M, Marx N, Reith S. Type 2 diabetes mellitus is associated with a lower fibrous cap thickness but has no impact on calcification morphology: an intracoronary optical coherence tomography study. *Cardiovasc Diabetol.* (2017) 16(1): 152. doi: 10.1186/s12933-017-0635-2

7 Ong DS, Lee JS, Soeda T, Higuma T, Minami Y, Wang Z, et al. Coronary Calcification and Plaque Vulnerability: An Optical Coherence Tomographic Study. *Circ Cardiovasc Imaging.* (2016) 9(1): e003929. doi: 10.1161/CIRCIMAGING.115.003929

2 Supplementary Figures and Tables

2.1 Supplementary Figures

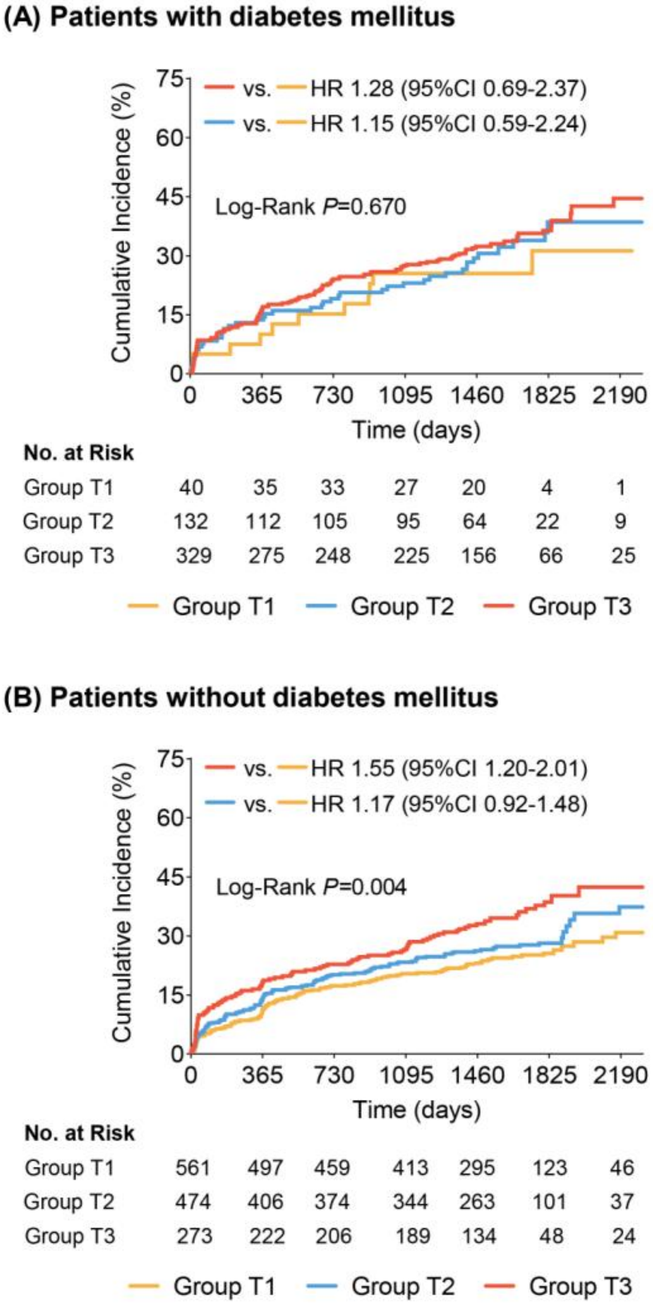

Figure S1. Kaplan-Meier curves for MACCE after stratification by diabetes status.

Cumulative incidence curves for MACCE in patients with diabetes mellitus **(A)** and without diabetes mellitus **(B)**. The The interaction *P*-value between TyG index and diabetes subgroup was 0.022. MACCE = major adverse cardiovascular and cerebrovascular events.

2.2 Supplementary Tables

Table S1. Proportion of dyslipidemia and medication use prior to hospitalization.

Variables	Group T1 (n=610)	Group T2 (n=611)	Group T3 (n=610)	*P*-value
Dyslipidemia	258 (42.3)	393 (64.3)	566 (92.8)	<0.001
TC≥220mg/dL	61 (10.0)	127 (20.8)	139 (22.8)	<0.001
TG≥150 mg/dL	6 (1.0)	170 (27.8)	484 (79.3)	<0.001
LDL≥140mg/dL	114 (18.7)	159 (26.0)	129 (21.1)	0.007
HDL≤40mg/dL	89 (14.6)	121 (19.8)	207 (33.9)	<0.001
Statin use prior to admission	61 (10.0)	73 (11.9)	94 (15.4)	0.015
Glucose-lowering therapy prior to admission	7 (1.1)	30 (4.9)	110 (18.0)	<0.001

HDL-C = high-density lipoprotein cholesterol; LDL-C = low-density lipoprotein cholesterol; TC = total cholesterol; TG = triglyceride.

Table S2. The correlation between TyG index and clinical risk factors.

Variables	Correlation coefficient (r)	*P* value
Age (years)	-0.112 [a]	<0.001
BMI (kg/m2)	0.215 [a]	<0.001
TC (mg/dL)	0.247 [a]	<0.001
TG (mg/dL)	0.851 [b]	<0.001
LDL-C (mg/dL)	0.047 [a]	0.047
HDL-C (mg/dL)	-0.298 [a]	<0.001
FBG (mg/dL)	0.592 [a]	<0.001
HbA1c (%)	0.519 [a]	<0.001
hsCRP (mg/L)	0.126 [b]	<0.001
eGFR (mL/min/1.73 m^2)	0.016 [b]	0.511
Urea nitrogen (μmol/L)	-0.006 [b]	0.792

BMI = body mass index; eGFR = estimated glomerular filtration rate; FBG = fasting blood glucose; HbA1c = glycated hemoglobin c; HDL-C = high-density lipoprotein cholesterol; hs-CRP = high-sensitive C-reactive protein; LDL-C = low-density lipoprotein cholesterol; TC = total cholesterol; TG = Triglyceride; TyG index = triglyceride glucose index.

[a]Pearson correlation analysis

[b]Spearman correlation analysis

Table S3. OCT analysis of calcification (calcium level).

Variables	Group T1 (n=611)	Group T2 (n=574)	Group T3 (n=501)	*P*-value	P^*-value		
					T1 vs. T2	T1 vs. T3	T2 vs. T3
Calcium length, mm	2.6 (1.4-5.1)	2.2 (1.1-4.0)	2.0 (1.1-4.0)	0.073	NA	NA	NA
Mean calcium arc, ◦	60.5 (40.2-88.4)	56.0 (34.8-83.9)	58.6 (37.5-88.0)	0.202	NA	NA	NA
Maximal calcium arc, ◦	80.7 (48.5-121.2)	71.6 (41.2-117.0)	72.4 (45.1-121.5)	0.374	NA	NA	NA
Maximal calcium arc >180°	68 (11.1)	62 (10.8)	59 (11.8)	0.894	NA	NA	NA
Calcium index	152.4 (56.2-414.0)	116.7 (48.9-316.0)	108.2 (49.2-307.8)	0.177	NA	NA	NA
Maximal calcium thickness, μm	750.0 (530.0-1040.0)	665.0 (442.5-980.0)	650.0 (430.0-930.0)	<0.001	0.003	<0.001	0.568
Maximal calcium thickness >500	467 (76.4)	388 (67.6)	338 (67.5)	0.006	0.009	0.006	0.976
Mean calcium depth, μm	165.0 (105.0-252.5)	184.5 (106.0-305.0)	180.0 (110.0-307.0)	0.174	NA	NA	NA
Minimal calcium depth, μm	70.0 (30.0-190.0)	100.0 (40.0-230.0)	100.0 (40.0-230.0)	0.029	0.027	0.025	0.962
Minimal calcium depth <100 μm	359 (58.8)	286 (49.8)	245 (48.9)	0.008	0.010	0.009	0.784
Minimal calcium depth <65 μm	273 (44.7)	222 (38.7)	191 (38.1)	0.073	NA	NA	NA
Mean calcium area, mm^2	0.8 (0.4-1.4)	0.6 (0.3-1.2)	0.6 (0.3-1.3)	0.033	0.079	0.010	0.537
Maximal calcium area, mm^2	1.2 (0.5-2.3)	0.9 (0.4-2.0)	0.9 (0.4-2.1)	0.077	NA	NA	NA
**Calcification type**							
Microcalcification	28 (4.6)	41 (7.1)	28 (5.6)	0.278	NA	NA	NA
Spotty calcification	280 (45.8)	284 (49.5)	252 (50.3)	0.366	NA	NA	NA
Macrocalcification	303 (49.6)	249 (43.4)	221 (44.1)	0.119	NA	NA	NA

Values are median (IQR). A *P*-value <0.05 or P^*-value <0.017 was considered statistically significant. OCT = optical coherence tomography.

Table S4. Multivariate regression analysis of the TyG index for the association culprit plaque characteristics.

Variables	OR	95%CI	*P*-value
**Plaque rupture**			
TyG index			
Group T1	1 (Reference)		
Group T2	1.39	1.06-1.82	0.018
Group T3	1.51	1.05-2.16	0.025
Age	1.04	1.03-1.05	<0.001
BMI	1.06	1.02-1.10	0.001
Current smokers	0.76	0.60-0.97	0.029
**TCFA**			
TyG index			
Group T1	1 (Reference)		
Group T2	1.27	0.98-1.64	0.069
Group T3	1.10	0.78-1.54	0.601
Age	1.03	1.02-1.04	<0.001
BMI	1.04	1.00-1.07	0.025
HDL	0.99	0.98-0.99	0.036
**Macrophage**			
TyG index			
Group T1	1 (Reference)		
Group T2	0.91	0.60-1.37	0.646
Group T3	0.92	0.51-1.69	0.794
Age	1.04	1.02-1.06	<0.001
HDL	0.98	0.96-0.99	0.017
**Cholesterol crystals**			
TyG index			
Group T1	1 (Reference)		
Group T2	1.01	0.79-1.29	0.954
Group T3	0.87	0.64-1.18	0.363
Age	1.02	1.01-1.03	0.002
Current smokers	0.77	0.62-0.95	0.016
Dyslipidemia	1.28	1.01-1.62	0.041
HDL	0.98	0.97-0.99	<0.001

	β-coefficient	*P*-value
**Lipid length**		
TyG index		
Group T1	1 (Reference)	
Group T2	0.023	0.423
Group T3	0.055	0.119
Age	0.085	0.002

Hypertension	0.056	0.028
Dyslipidemia	0.067	0.016
Uric nitrogen	0.061	0.018
**Lipid index**		
TyG index		
Group T1	1 (Reference)	
Group T2	0.024	0.423
Group T3	0.065	0.072
Age	0.073	0.007
Hypertension	0.054	0.032
Dyslipidemia	0.061	0.029
**Calcium length**		
TyG index		
Group T1	1 (Reference)	
Group T2	-0.056	0.181
Group T3	-0.027	0.553
Age	0.201	<0.001
Dyslipidemia	-0.107	0.008
**Maximal calcium thickness**		
TyG index		
Group T1	1 (Reference)	
Group T2	-0.101	0.019
Group T3	-0.035	0.512
Age	0.144	0.001
Dyslipidemia	-0.110	0.009

Values are OR (95%CI). Only results from the variables that were significant ($P<0.05$) and TyG index in the multivariate analysis are shown in the table. The covariates include age, sex, BMI, current smokers, hypertension, dyslipidemia, diabetes mellitus, CKD, previous MI, TC, LDL-C, HDL-C, HbA1c, and hs-CRP. BMI = body mass index; CKD = chronic kidney disease; CI = confidence interval; FCT = fibrous cap thickness; HbA1c = glycated hemoglobin c; HDL-C = high-density lipoprotein cholesterol; hs-CRP = high-sensitive C-reactive protein; LDL-C = low-density lipoprotein cholesterol; MI = myocardial infarction; OR = odds ratio; TC = total cholesterol; TCFA=thin cap fibroatheroma; TyG index = triglyceride glucose index.

Table S5. Medications use after discharge.

Variables	Group T1 (n=610)	Group T2 (n=611)	Group T3 (n=610)	*P*-value
Aspirin	601 (98.5)	603 (98.7)	597 (97.9)	0.489
P2Y12 inhibitor	603 (98.9)	606 (99.2)	601 (98.5)	0.516
DAPT	600 (98.4)	602 (98.5)	596 (97.7)	0.563
Statins	601 (98.8)	602 (98.9)	598 (98.0)	0.736
Atorvastatin	472 (77.4)	481 (78.7)	454 (74.4)	0.191
Rosuvastatin	126 (20.7)	116 (19.0)	142 (23.3)	0.178
Simvastatin	0 (0.0)	1 (0.2)	0 (0.0)	1.000
Fluvastatin	5 (0.8)	4 (0.7)	0 (0.0)	0.075
β-blockers	390 (63.9)	395 (64.6)	416 (68.2)	0.244
ACEI/ARB	289 (47.4)	322 (52.7)	320 (52.5)	0.110

Values are n (%). A *P*-value <0.05 was considered statistically significant. ACEI = angiotensin-converting enzyme inhibitor; ARB = angiotensin receptor blocker; DAPT = dual antiplatelet therapy.

Table S6. Univariate Cox proportional hazard model to predict MACCE.

Variables	**HR (95%CI)**	***P* value**
TyG index		
Group T1	1 (reference)	
Group T2	1.19 (0.96-1.48)	0.115
Group T3	1.51 (1.22-1.86)	<0.001
Age	1.01 (1.00-1.02)	0.002
Female	1.22 (1.02-1.47)	0.034
Body mass index	1.02 (1.00-1.05)	0.044
Current smoking	0.80 (0.68-0.95)	0.009
Hypertension	1.55 (1.31-1.84)	<0.001
Dyslipidemia	1.10 (0.92-1.32)	0.311
Diabetes mellitus	1.23 (1.03-1.48)	0.026
Prior myocardial infarction	1.78 (1.30-2.43)	<0.001
Chronic kidney disease	1.36 (1.09-1.68)	0.005
TC	0.99 (0.99-1.00)	0.408
LDL-C	1.00 (0.99-1.00)	0.757
HDL-C	0.99 (0.98-0.99)	0.009
HbA1c	1.06 (1.00-1.13)	0.042
hs-CRP	1.01 (1.00-1.03)	0.039
Urea nitrogen	1.06 (1.02-1.10)	0.004
Aspirin	0.61 (0.32-1.18)	0.142
P2Y12 inhibitor	0.70 (0.29-1.70)	0.433
Statins	0.69 (0.34-1.39)	0.296
β-blockers	0.96 (0.80-1.14)	0.610
ACEI/ARB	1.00 (0.85-1.19)	0.960
Multivessel disease	1.64 (1.33-2.02)	<0.001
Culprit lesion vessel		
Left circumflex artery	1 (reference)	
Left anterior descending artery	0.85 (0.65-1.13)	0.263
Right coronary artery	0.99 (0.75-1.31)	0.945
Plaque rupture	1.14 (0.94-1.39)	0.176
TCFA	1.17 (0.98-1.40)	0.088
Macrophage	1.39 (1.00-1.94)	0.050
Microvessel	0.99 (0.84-1.18)	0.931
Cholesterol crystals	1.11 (0.94-1.32)	0.229
Thrombus	0.87 (0.55-1.38)	0.562
Calcification	1.16 (0.98-1.38)	0.078

Values are HR (95%CI). Values are HR (95%CI). HR = hazard ratio; MACCE = major adverse cardiovascular and cerebrovascular events; TCFA = thin cap fibroatheroma. Other abbreviations as shown in Tables S2 and S5.

Table S7. The incremental predictive value of the TyG index for MACCE.

	C-statistic (95% CI)	*P*-value	Continuous NRI (95% CI)	*P*-value	IDI (95% CI)	*P*-value
**Overall**						
Baseline risk model	0.581 (0.555-0.606)	Ref	Ref		Ref	
Baseline risk model+ TyG index	0.595 (0.570-0.620)	0.038	0.230 (0.132-0.340)	<0.001	0.008 (0.004-0.012)	<0.001
**Patients who did not receive lipid- or glucose-lowering therapy prior to admission**						
Baseline risk model	0.576 (0.562-0.591)	Ref	Ref		Ref	
Baseline risk model+ TyG index	0.590 (0.576-0.605)	0.043	0.202 (0.091-0.319)	<0.001	0.008 (0.004-0.013)	<0.001

Baseline risk model included age, gender, hypertension, dyslipidemia, diabetes mellitus, previous myocardial infarction, TC, LDL-C, HDL, HbA1c, hs-CRP. IDI = integrated discrimination improvement; NRI = net reclassification improvement. Other abbreviations as shown in Tables S2 and S6.

Table S8. OCT findings of culprit lesions in patients without prior lipid/glucose-lowering therapy.

	Group T1 (n=498)	Group T2 (n=498)	Group T3 (n=498)	*P*-value	P^*-value		
					T1 vs. T2	T1 vs. T3	T2 vs. T3
**Culprit lesion type**							
Plaque rupture	315 (63.3)	359 (72.1)	389 (78.1)	<0.001	0.003	<0.001	0.028
Plaque erosion	176 (35.3)	131 (26.3)	107 (21.5)	<0.001	0.002	<0.001	0.075
Calcified nodule	7 (1.4)	7 (1.4)	2 (0.4)	0.206	NA	NA	NA
Lesion length, mm	18.3 (14.0-24.0)	18.5 (15.0-23.8)	19.0 (14.8-25.0)	0.509	NA	NA	NA
MLA, mm^2	1.2 (0.9-1.6)	1.1 (0.9-1.5)	1.1 (0.9-1.5)	0.129	NA	NA	NA
AS, %	82.2 (76.5-87.0)	83.7 (78.3-87.4)	83.6 (75.9-87.8)	0.025	0.006	0.134	0.278
Lipid plaque	439 (88.2)	455 (91.4)	463 (93.0)	0.027	0.094	0.009	0.345
Lipid length, mm	10.8 (6.0-15.4)	11.0 (7.0-15.5)	12.0 (8.0-17.4)	<0.001	0.214	<0.001	0.003
Mean lipid arc, °	163.9 (132.8-201.4)	171.8 (137.7-209.2)	173.5 (143.1-210.8)	0.025	0.042	0.01	0.585
Maximum lipid arc, °	304.6 (217.2-360.0)	332.1 (235.2-360.0)	360.0 (239.6-360.0)	0.038	0.099	0.012	0.389
Lipid index	1928.7 (1119.3-2986.4)	1990.4 (1260.8-2963.0)	2282.7 (1433.2-3342.9)	<0.001	0.228	<0.001	0.005
Minimal FCT, µm	60.0 (47.0-80.0)	57.0 (43.3-70.0)	57.0 (43.0-70.0)	0.237	NA	NA	NA
Lipid-rich plaque	437 (87.8)	453 (91.0)	461 (92.6)	0.031	0.100	0.011	0.356
TCFA	271 (54.4)	313 (62.9)	329 (66.1)	0.001	0.007	<0.001	0.290
Macrophage	436 (87.6)	451 (90.6)	455 (91.5)	0.093	NA	NA	NA
Microvessel	197 (39.6)	195 (39.2)	197 (39.7)	0.983	NA	NA	NA
Cholesterol crystals	231 (46.4)	250 (50.2)	268 (53.9)	0.059	NA	NA	NA
Thrombus	484 (97.2)	480 (96.4)	479 (96.2)	0.653	NA	NA	NA
Calcification	215 (43.2)	201 (40.4)	193 (38.8)	0.357	NA	NA	NA
Spotty calcification	147 (29.5)	146 (29.3)	142 (28.5)	0.934	NA	NA	NA
Superficial-65 calcification	160 (32.1)	118 (23.7)	121 (24.3)	0.004	0.003	0.006	0.824
Superficial-100 calcification	195 (39.2)	145 (29.1)	149 (29.9)	0.001	0.001	0.002	0.781
Total calcification number	2.0 (1.0-3.0)	2.0 (1.0-3.0)	2.0 (1.0-3.0)	0.063	NA	NA	NA

Calcium length, mm	7.0 (3.7-12.2)	5.8 (2.6-8.9)	5.4 (2.6-9.8)	0.010	0.005	0.018	0.720
Mean calcium arc, ◦	71.2 (49.7-95.2)	64.2 (45.1-92.8)	67.7 (43.0-91.7)	0.242	NA	NA	NA
Maximal calcium arc, ◦	111.8 (76.0-166.8)	107.0 (66.4-169.6)	105.3 (63.5-167.8)	0.422	NA	NA	NA
Calcium index	499.0 (193.0-1121.8)	363.3 (133.4-714.4)	345.8 (130.6-911.0)	0.019	0.010	0.028	0.815
Maximal calcium thickness, µm	980.0 (740.0-1220.0)	840.0 (600.0-1130.0)	820.0 (587.5-1112.5)	<0.001	0.001	0.001	0.755
Mean calcium depth, µm	167.0 (115.0-229.0)	186.0 (125.0-280.0)	189.0 (119.8-271.2)	0.087	NA	NA	NA
Minimal calcium depth, µm	40.0 (20.0-70.0)	50.0 (23.0-123.0)	43.0 (20.0-111.8)	0.019	0.004	0.112	0.333
Mean calcium area, mm²	1.0 (0.5-1.7)	0.8 (0.4-1.3)	0.8 (0.4-1.4)	0.012	0.008	0.016	0.819
Maximal calcium area, mm²	2.0 (1.1-3.5)	1.5 (0.7-3.0)	1.5 (0.7-3.1)	0.008	0.007	0.009	0.955

Values are n (%) or median (IQR). A *P*-value <0.05 or P^*-value <0.017 was considered statistically significant. AS = area stenosis; FCT = fibrous cap thickness; MLA = minimal lumen area; OCT = optical coherence tomography; RLA = reference lumen area; TCFA = thin cap fibroatheroma.

Table S9. Univariate and multivariate logistic regression analysis to predict plaque rupture in patients without prior lipid/glucose-lowering therapy.

	Univariate		Multivariate	
	OR (95%CI)	*P* value	OR (95%CI)	*P* value
TyG index				
Group T1	1 (Reference)		1 (Reference)	
Group T2	1.50 (1.15-1.96)	0.003	1.51 (1.12-2.03)	0.007
Group T3	2.07 (1.57-2.74)	<0.001	1.77 (1.20-2.62)	0.004
Age	1.03 (1.02-1.04)	<0.001	1.04 (1.03-1.05)	<0.001
Female	1.37 (1.05-1.78)	0.020	1.06 (0.77-1.45)	0.743
BMI	1.05 (1.02-1.08)	0.003	1.07 (1.03-1.11)	0.001
Current smokers	0.49 (0.39-0.63)	<0.001	0.74 (0.56-0.97)	0.028
Hypertension	1.49 (1.19-1.87)	0.001	1.04 (0.80-1.35)	0.783
Dyslipidemia	1.53 (1.22-1.93)	<0.001	1.15 (0.86-1.55)	0.345
Diabetes mellitus	1.67 (1.24-2.24)	0.001	0.95 (0.57-1.57)	0.835
Chronic kidney disease	1.48 (1.06-2.07)	0.022	0.82 (0.55-1.23)	0.343
Previous myocardial infarction	1.01 (0.48-2.13)	0.971		
TC	1.01 (1.00-1.01)	0.002	1.00 (0.99-1.01)	0.294
LDL	1.00 (1.00-1.01)	0.013	1.00 (0.99-1.01)	0.998
HDL	0.99 (0.98-1.00)	0.198		
HbA1c	1.22 (1.09-1.36)	<0.001	1.06 (0.89-1.26)	0.507
hs-CRP	1.00 (0.98-1.03)	0.729		

Abbreviations as shown in Table S2.

Table S10. Univariate and multivariate Cox regression analysis to predict MACCE in patients without prior lipid/glucose-lowering therapy.

	HR (95%CI)		
	**Model 1**	**Model 2**	**Model 3**
**TyG index as a continuous variable**			
Per 1 unit increase	1.21 (1.06-1.38) **	1.19 (1.02-1.40) *	1.18 (1.01-1.39) *
Per 1 SD increase	1.14(1.04-1.25) **	1.13 (1.01-1.27) *	1.12 (1.00-1.26) *
**TyG index as a nominal variable**			
Group T1	1 (Reference)	1 (Reference)	1 (Reference)
Group T2	1.12 (0.87-1.43)	1.14 (0.88-1.46)	1.12 (0.87-1.44)
Group T3	1.36 (1.08-1.73) *	1.34 (1.02-1.75) *	1.32 (1.00-1.73) *
P for trend	0.006	0.031	0.042

Values are HR (95%CI). $^{*}P <0.05$ and $^{**}P <0.001$. Model 1 unadjusted; Model 2 adjusted for age, sex, BMI, current smokers, hypertension, dyslipidemia, diabetes mellitus, chronic kidney disease, previous myocardial infarction, TC, LDL-C, HDL-C, HbA1c, hs-CRP, aspirin, P2Y12 inhibitor, statins, β-blockers, ACEI/ARB; Model 3 adjusted for all factors in model 2 plus culprit lesion vessel, multivessel disease, plaque rupture, TCFA, macrophage, microvessels, cholesterol crystals, thrombus, and calcification. CI = confidence interval; HR = hazards ratio; MACCE = major adverse cardiovascular and cerebrovascular events. Other abbreviations as shown in Tables S2 and S5.

Table S11. Culprit plaque OCT features after stratification by diabetes status.

	Patients with diabetes mellitus				Patients without diabetes mellitus			
	Group T1 (n=40)	Group T2 (n=134)	Group T3 (n=334)	*P*-value	Group T1 (n=570)	Group T2 (n=477)	Group T3 (n=276)	*P*-value
**Culprit lesion type**								
Plaque rupture	30 (75.0)	107 (79.9)	248 (74.3)	0.439	370 (64.9)	337 (70.6)	218 (79.0)	<0.001
Plaque erosion	10 (25.0)	26 (19.4)	83 (24.9)	0.440	191 (33.5)	134(28.1)	55 (19.9)	<0.001
Calcified nodule	0 (0.0)	1 (0.7)	3 (0.9)	1.000	9 (1.6)	5 (1.0)	3 (1.1)	0.775
Lesion length, mm	19.7 (13.8-23.2)	19.1 (15.0-27.0)	19.0 (14.1-25.1)	0.639	18.0 (14.0-24.0)	18.4 (14.6-24.0)	19.4 (15.0-25.0)	0.242
MLA, mm²	1.3 (1.0-1.9)	1.1 (0.9-1.4)	1.1 (0.9-1.5)	0.044	1.2 (0.9-1.6)	1.1 (0.9-1.5)	1.1 (0.9-1.5)	0.179
AS, %	81.0 (73.2-88.7)	83.3 (78.1-87.4)	82.6 (75.2-87.3)	0.256	82.6 (76.9-87.0)	83.7 (78.2-87.8)	83.8 (77.8-88.2)	0.030
Lipid plaque	38 (95.0)	130 (97.0)	315 (94.3)	0.474	504 (88.4)	428 (89.7)	260 (94.2)	0.029
Lipid length, mm	11.6 (9.1-16.4)	11.6 (8.0-16.7)	12.0 (8.3-17.4)	0.775	10.7 (6.0-15.0)	11.0 (7.0-15.0)	11.6 (7.7-17.7)	0.005
Mean lipid arc, °	187.3 (148.9-213.4)	173.7 (140.8-206.6)	164.2 (132.7-205.6)	0.268	164.3 (132.3-201.2)	171.4 (136.3-211.2)	177.8 (148.7-217.4)	<0.001
Maximum lipid arc, °	338.9 (272.9-360.0)	360.0 (227.1-360.0)	298.7 (225.6-360.0)	0.310	303.7 (217.4-360.0)	318.9 (235.4-360.0)	360.0 (261.7-360.0)	0.001
Lipid index	2170.2 (1647.5-2915.3)	2017.7 (1223.3-3100.3)	2139.9 (1412.7-3271.1)	0.809	1893.9 (1076.9-3012.2)	1995.0 (1243.2-2969.9)	2414.3 (1428.8-3521.9)	0.001
Minimal FCT, μm	50.0 (40.0-60.0)	56.8 (40.8-60.0)	57.0 (46.8-75.0)	0.060	60.0 (47.0-80.0)	57.0 (43.0-70.0)	57.0 (41.5-70.0)	0.169
Lipid-rich plaque	38 (95.0)	130 (97.0)	313 (93.7)	0.403	501 (87.9)	426 (89.3)	259 (93.8)	0.028
TCFA	31 (77.5)	99 (73.9)	218 (65.3)	0.086	317 (55.6)	296 (62.1)	187 (67.8)	0.002
Macrophage	38 (95.0)	127 (94.8)	316 (94.9)	0.998	500 (87.7)	422 (88.5)	257 (93.1)	0.052
Microchannels	17 (42.5)	49 (36.6)	139 (41.9)	0.553	226 (39.6)	186 (39.0)	110 (40.0)	0.958
Cholesterol crystals	23 (57.5)	88 (65.7)	199 (59.8)	0.439	270 (47.4)	228 (47.8)	145 (52.5)	0.336

Thrombus	38 (95.0)	129 (96.3)	323 (96.7)	0.851	501 (87.9)	426 (89.3)	259 (93.8)	0.028
Calcification	27 (67.5)	71 (53.0)	155 (46.4)	0.029	245 (43.0)	181 (37.9)	105 (38.0)	0.185
Spotty calcification	19 (47.5)	53 (39.6)	115 (34.4)	0.201	169 (29.6)	132 (27.7)	84 (30.4)	0.673
Superficial-65	18 (45.0)	46 (34.3)	98 (29.3)	0.104	177 (31.1)	101 (21.2)	65 (23.6)	0.001
Superficial-100	20 (50.0)	55 (41.0)	119 (35.6)	0.153	212 (37.2)	125 (26.2)	80 (29.0)	<0.001
Total calcification number	2.0 (1.0-2.5)	2.0 (1.0-3.0)	2.0 (1.0-3.0)	0.408	2.0 (1.0-3.0)	2.0 (1.0-3.0)	2.0 (1.0-3.0)	0.063
Calcium length, mm	5.5 (2.1-9.2)	5.5 (3.7-11.2)	5.6 (3.0-9.4)	0.513	7.0 (3.5-12.4)	5.1 (2.1-8.5)	5.5 (2.5-10.6)	0.001
Mean calcium arc, ◦	61.4 (49.2-90.2)	61.6 (46.7-101.8)	69.8 (49.6-94.7)	0.538	71.0 (49.6-95.5)	62.7 (42.5-89.6)	62.8 (43.0-93.0)	0.072
Maximal calcium arc, ◦	95.5 (70.7-116.8)	103.1 (80.1-178.9)	109.3 (69.6-170.0)	0.338	112.5 (76.1-169.6)	97.4 (53.4-156.7)	106.2 (60.6-176.8)	0.073
Calcium index	217.8 (152.4-832.7)	357.3 (180.9-912.7)	375.5 (174.3-815.1)	0.592	501.5 (189.4-1168.5)	348.7 (103.9-686.1)	351.6 (107.3-991.4)	0.002
Maximal calcium thickness, µm	930.0 (690.0-1055.0)	940.0 (645.0-1135.0)	835.0 (655.2-1092.5)	0.687	980.0 (740.0-1240.0)	810.0 (560.0-1120.0)	880.0 (610.0-1170.0)	<0.001
Mean calcium depth, µm	190.0 (128.0-231.5)	184.0 (127.0-257.0)	180.5 (116.8-260.2)	0.821	169.0 (120.0-239.0)	190.0 (125.0-283.0)	200.0 (127.0-310.0)	0.099
Minimal calcium depth, µm	30.0 (20.0-140.0)	47.0 (30.0-88.5)	35.0 (20.0-90.8)	0.188	40.0 (20.0-80.0)	57.0 (20.0-140.0)	53.0 (23.0-130.0)	0.031
Mean calcium area, mm²	0.8 (0.6-1.3)	0.9 (0.4-1.5)	0.8 (0.5-1.3)	0.765	1.0 (0.5-1.6)	0.8 (0.3-1.2)	1.0 (0.4-1.6)	0.002
Maximal calcium area, mm²	1.5 (1.3-2.5)	1.7 (0.8-3.7)	1.6 (0.8-3.0)	0.847	2.0 (1.1-3.5)	1.4 (0.6-2.7)	1.9 (0.7-3.4)	0.001

Values are n (%) or median (IQR). A *P*-value <0.05 was considered statistically significant. Abbreviations as shown in Tables S2.

Table S12. Multivariate logistic regression analysis for culprit plaque characteristics in patients without diabetes mellitus.

Variables	OR	95%CI	*P*-value
**Plaque rupture**			
TyG index			
Group T1	1 (Reference)		
Group T2	1.31	0.98-1.76	0.072
Group T3	2.12	1.37-3.27	0.001
Age	1.04	1.03-1.06	<0.001
BMI	1.08	1.03-1.12	<0.001
Current smokers	0.72	0.54-0.96	0.024
**TCFA**			
TyG index			
Group T1	1 (Reference)		
Group T2	1.25	0.95-1.64	0.108
Group T3	1.41	0.95-2.09	0.089
Age	1.03	1.01-1.04	<0.001
HDL	0.99	0.97-0.99	0.021
**Macrophage**			
TyG index			
Group T1	1 (Reference)		
Group T2	0.77	0.51-1.18	0.228
Group T3	1.00	0.51-1.96	0.999
Age	1.04	1.03-1.06	<0.001
Dyslipidemia	1.60	1.01-2.52	0.045
HDL	0.98	0.96-0.99	0.040
	**β-coefficient**		***P*-value**
**Lipid length**			
TyG index			
Group T1	1 (Reference)		
Group T2	0.000		0.998
Group T3	0.052		0.120
Age	0.089		0.006
BMI	0.062		0.044
Hypertension	0.059		0.043
Dyslipidemia	0.087		0.006
**Lipid index**			
TyG index			
Group T1	1 (Reference)		
Group T2	0.019		0.571

Group T3	0.079	0.027
Female	-0.067	0.036
**Calcium length**		
TyG index		
Group T1	1 (Reference)	
Group T2	-0.084	0.076
Group T3	-0.006	0.898
Age	0.166	0.001
Dyslipidemia	-0.133	0.007
**Maximal calcium thickness**		
TyG index		
Group T1	1 (Reference)	
Group T2	-0.138	0.005
Group T3	-0.054	0.311
Dyslipidemia	-0.104	0.038

Values are OR (95%CI). Only results from the variables that were significant ($P<0.05$) and TyG index in the multivariate analysis are shown in the table. The covariates include age, sex, BMI, current smokers, hypertension, dyslipidemia, CKD, previous MI, TC, LDL-C, HDL-C, HbA1c, and hs-CRP. Abbreviations as shown in Table S4.

Table S13. Univariate and multivariate Cox regression analysis for MACCE in patients without diabetes mellitus.

	HR (95%CI)		
	**Model 1**	**Model 2**	**Model 3**
TyG index as a continuous variable			
Per 1 unit increase	1.36 (1.15-1.61) **	1.38 (1.14-1.65) *	1.35 (1.12-1.62) *
Per 1 SD increase	1.25 (1.11-1.41) **	1.26 (1.10-1.44) *	1.24 (1.09-1.42) *
TyG index as a nominal variable			
Group T1	1 (Reference)	1 (Reference)	1 (Reference)
Group T2	1.17 (0.92-1.48)	1.17 (0.91-1.50)	1.17 (0.91-1.51)
Group T3	1.55 (1.20-2.01) **	1.55 (1.16-2.06) *	1.48 (1.11-1.98) *
P for trend	<0.001	0.001	0.001

Values are HR (95%CI). $^{*}P$ <0.05 and $^{**}P$ <0.001. Model 1 unadjusted; Model 2 adjusted for age, sex, BMI, current smokers, hypertension, dyslipidemia, diabetes mellitus, chronic kidney disease, previous MI, TC, LDL-C, HDL-C, HbA1c, hs-CRP, aspirin, P2Y12 inhibitor, statins, β-blockers, ACEI/ARB; Model 3 adjusted for all factors in model 2 plus culprit lesion vessel, multivessel disease, plaque rupture, TCFA, macrophage, microvessels, cholesterol crystals, thrombus, and calcification. CI = confidence interval; HR = hazards ratio; MACCE = major adverse cardiovascular and cerebrovascular events. Other abbreviations as shown in Tables S2 and S5.
